# Escape and evasion: when immunosurveillance of senescent cells goes wrong

**DOI:** 10.3389/fgene.2026.1882818

**Published:** 2026-07-07

**Authors:** Elina Shakur, Abraham Jacobs, Rituparna Ghosh, Matthew J. Yousefzadeh

**Affiliations:** 1 Columbia Center for Human Longevity, Columbia University Medical Center, New York, NY, United States; 2 Columbia Center for Translational Immunology, Columbia University Medical Center, New York, NY, United States; 3 Department of Medicine, Columbia University Medical Center, New York, NY, United States

**Keywords:** cellular senescence, immunosenescence, immunosurveillance, inflammaging, SASP

## Abstract

Aging involves molecular changes that can give rise to different cell fates, one of those being cellular senescence. Senescent cells stably arrest in the cell cycle and play important roles in physiological processes and can act in a tumor-suppressive manner. However, senescent cells accumulate throughout the body with both chronological and biological aging, promoting chronic inflammation and tissue dysfunction. One of the features of senescent cells is their ability to adopt a secretory phenotype, which can act as a chemotactic gradient to attract immune cells. These infiltrating immune cells are capable of recognizing senescent cells and targeting them for destruction, thus maintaining a balance between senescent cell generation and elimination. Unfortunately, with age, the immune system undergoes changes that alter functional capacity, referred to as immunosenescence. Immunosenescence impacts both innate and adaptive immune cells, impairing their protective functions, like immunosurveillance, or causing them to adopt a hyperinflammatory phenotype, which may further enhance senescent cell burden. These age-related changes in immune function can compromise immunosurveillance, further exacerbating senescent cell burden and its effects. Additionally, senescent cells themselves can modulate markers on their cell surface that make detection by immune cells more difficult and allow them to escape immune clearance. The role of the immune system in limiting senescent cell burden to maintain homeostasis and how immunosurveillance is compromised with age is explored. Furthermore, mechanisms by which senescent cells evade immunosurveillance and potential strategies to restore age-related deficits in immune cell-mediated clearance of senescent cells are also discussed.

## Introduction

As we age, our bodies undergo a complex and interconnected series of physiological changes, that are governed by underlying molecular pathways referred to as the Hallmarks of Aging (Lopez-Otin et al., 2013; Lopez-Otin et al., 2023). One of these hallmarks, cellular senescence induces a cell fate whereby cells have impaired proliferation yet are still metabolically active and are causal agents of aging (Zhang et al., 2022b). Under healthy conditions, senescent cells can be beneficial and transient, most notably aiding in embryonic development, wound healing and tumor suppression in a context-dependent manner (de Magalhaes, 2024). However with aging, senescent cells accumulate with age in tissues to disrupt homeostasis, impair regeneration, and negatively impact both healthspan and lifespan (Zhang et al., 2022b). In the context of normal physiology and age-or biological stress-induced formation, senescent cells have immunogenic properties that are permissive for the recruitment of immune cells in order to facilitate clearance (Majewska and Krizhanovsky, 2025b; Wang and Nakanishi, 2025). Normally, acute senescent cells are cleared by the immune system; however, with age and the onset of immunosenescence (immune aging) senescent cells can accumulate and secrete inflammatory factors that can help them escape immune surveillance, leading to chronic inflammation and age-related disease (Majewska and Krizhanovsky, 2025b; Jang et al., 2026). This review explores why cellular senescence occurs and the distinct characteristics of senescence. It highlights the mechanisms our immune system uses to remove these cells and how, with age, immunosenescence, and certain mechanisms used by senescent cells to evade surveillance.

## Senescent cells and their role in inflammaging

Senescent cells can arise from any number of endogenous or exogenous forms of stress (Roger et al., 2021; Huang et al., 2022; Reimann et al., 2024). However, the stability and irreversible fate of senescent cells depend on both cell type and the senescence-inducing stimulus. While senescence has traditionally been defined as a stable and irreversible cell-cycle arrest, accumulating evidence suggests that this state can be reversible in certain contexts (Reimann et al., 2024). For example, oncogene-induced senescence (OIS) is increasingly recognized as an unstable barrier, with senescent cells capable of re-entering the cell cycle under specific conditions. Recent work has identified transcriptional mechanisms regulated by AP-1 and POU2F2 that can facilitate senescence escape (Martinez-Zamudio et al., 2020; Martinez-Zamudio et al., 2023). In contrast, other forms of senescence, such as replicative senescence driven by telomere shortening, are generally considered more durable and likely irreversible (Fumagalli et al., 2014).

**FIGURE 1:**
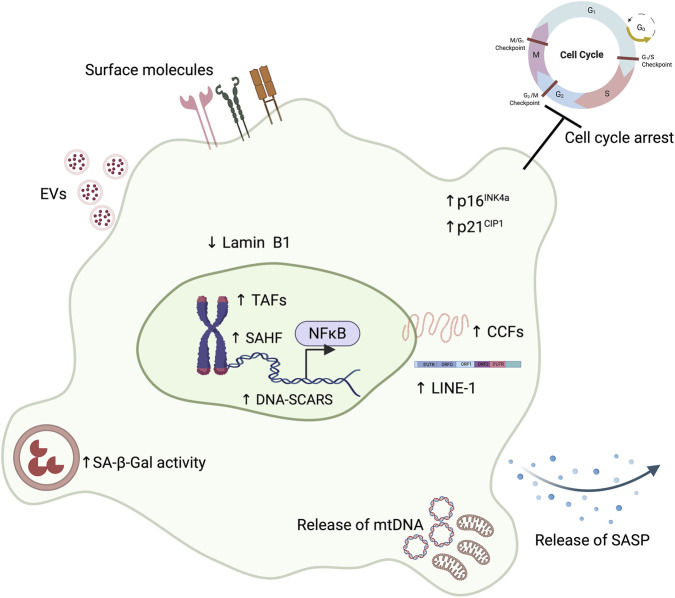
Features of senescent cells. Senescent cells display diverse features spanning enzymatic activity (senescence-associated β-galactosidase; SA-β-Gal), molecular changes (increased expression of cyclin-dependent kinase inhibitors such as p16^INK4a^ and p21^CIP1^ that enforce cell-cycle arrest, altered nuclear architecture, chromatin remodeling, and the appearance of cytoplasmic nucleic acids derived from the nucleus and mitochondria), morphological alterations (cell enlargement and a flattened/irregular appearance), changes in cell-surface protein presentation, and secretory remodeling consistent with the senescence-associated secretory phenotype (SASP). CCFs, cytoplasmic chromatin fragments; DNA-SCARS, DNA segments with chromatin alterations reinforcing senescence; EV, extracellular vesicles; LINE-1, long interspersed nuclear element 1; mtDNA, mitochondrial DNA; SAHF, senescence-associated heterochromatin foci; SASP, senescence-associated secretory phenotype; TAFs, telomere-associated DNA damage foci. Figure made with BioRender.

While no universal marker for senescent cells exists, they develop a signature marked by molecular and phenotypic changes including morphological changes, expression of cyclin-dependent kinase inhibitors (p16^INK4a^, and p21^CIP1^), loss of nuclear structure and release of chromatin fragments into the cytoplasm, DNA damage signaling, enzymatic changes including senescence-associated β-galactosidase (SA-β-Gal) activity at pH 6.0, and the adoption of a secretory phenotype (Figure 1) (Dimri et al., 1995; Coppe et al., 2010; Freund et al., 2012; Jackstadt et al., 2013; Sharpless and Sherr, 2015; Dou et al., 2017; Miller et al., 2021; Feng et al., 2025). While many features of senescent cells exist the development of the senescence-associated secretory phenotype (SASP) is important for their impact on aging as well as their detection and clearance. The SASP is comprised of pro-inflammatory cytokines, chemokines, extracellular vesicles, growth factors, lipids, nucleic acids, and proteases (Wang et al., 2024). Although the exact composition varies depending on the cell type and trigger, the overall effect is the induction of localized and potentially systemic inflammation. Through these signals, senescent cells can recruit immune cells and even induce senescence in nearby cells in what is known as paracrine senescence (Huang et al., 2022; Wang et al., 2024). Elimination of senescent cells using genetic, pharmacologic, or biologic approaches has been shown to improve overall health and drugs targeting senescent cells are currently being explored in clinical trials for multiple age-related chronic diseases (Zhang et al., 2022a; Khosla et al., 2025).

The accumulation of senescent cells over time plays a central role in the development of inflammaging, a chronic, low-grade inflammatory state associated with aging (Franceschi et al., 2000). While senescence initially serves as a protective response to cellular stress, the paradoxical long-term effect of these cells results in tissue dysfunction (Zhang et al., 2022b; De Magalhaes, 2024). This context-dependent nature of senescent cells is impacted by shifts in the composition of the SASP from immunogenic to hyperinflammatory (Wang et al., 2024). SASP has been shown to induce both local and systemic inflammation and is a major contributor to inflammaging, even in the absence of infection (Wang et al., 2024). In addition to causing tissue dysfunction, SASP factors can reinforce senescence in an autocrine manner and induce senescence in neighboring cells through paracrine signaling (Acosta et al., 2013). This establishes a feedback loop in which inflammation promotes further senescence, and the resulting senescent cells reinforce and sustain chronic inflammation.

Although the immune system plays an important role in recognizing and clearing senescent cells, this process becomes less efficient with age. Under normal conditions, senescent cells are cleared through immune-mediated mechanisms (Majewska and Krizhanovsky, 2025b; Wang and Nakanishi, 2025). SASP secreted by senescent cells recruits immune cells, including macrophages, natural killer cells, and T lymphocytes, which facilitate their elimination (Wang et al., 2024). This process is essential for maintaining healthy tissue following events such as oncogenic stress. However, with aging, this clearance system becomes less efficient due to declining immune function, which impairs its ability to remove senescent cells (Ovadya et al., 2018; Majewska and Krizhanovsky, 2025b). In addition, senescent cells can evade immune detection through mechanisms such as expression of inhibitory ligands or disruption of immune recognition pathways (Majewska et al., 2024; Majewska and Krizhanovsky, 2025a). As a result, senescent cells accumulate in tissues and directly promoting chronic inflammation and disrupting tissue homeostasis and regeneration (Wrona et al., 2024).

## Immune-mediated clearance of senescent cells

**FIGURE 2:**
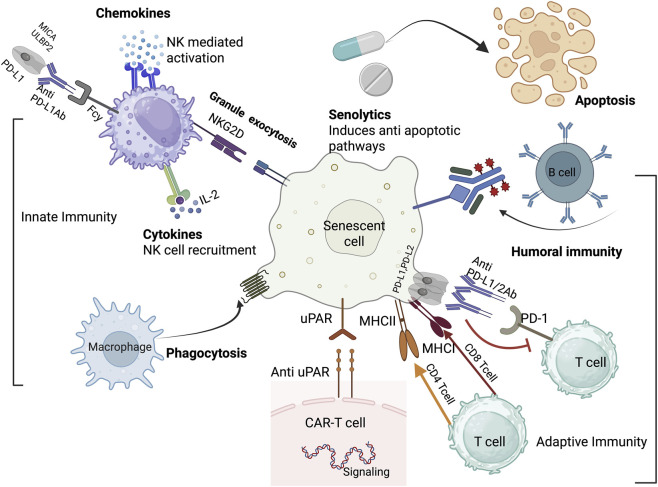
Immunosurveillance of senescent cells and their mechanisms of immune evasion. Senescent cells can be removed either by stimulating immune-mediated surveillance or by inducing apoptosis with senolytic agents. Senescent cells express immunogenic ligands—including MICA, ULBP2, MHC class I, and MHC class II—that are detected by immune effector populations such as NK cells, T cells, macrophages, and engineered CAR-T cells. In addition, B cells contribute to senescent-cell clearance through antibody-dependent (humoral) immune mechanisms.

As previously mentioned, SASP plays an important role in promoting anti-tumor immunity and maintaining tissue homeostasis via the clearance of neoplastic and senescent cells (Coppe et al., 2010). Senescent cells actively upregulate “find me” signals such as CXCL10, CCL5, and CCL2, they also upregulate surface stress ligands-particularly NKG2DL, a common ligand shared by cells that are stressed out (Soriani et al., 2009; Soriani et al., 2013). It has been shown that through the secretion of a mixture of cytokines and chemokines, senescent cells have the ability to reshape its microenvironment into a pro-immunogenic one. A study conducted using immune-competent liver cancer models discovered that senescent cells upregulated type II IFN (IFN-γ) receptors, thereby allowing a vigorous recruitment of immune cells (Chen et al., 2023). Furthermore, SASP factors have been shown to aid in wound healing as well (Borgoni et al., 2021). In acute cutaneous wounds, senescent fibroblasts secrete PDGF-AA to promote differentiation of non-senescent fibroblasts into myofibroblasts, which are essential in driving wound contraction and collagen production (Demaria et al., 2014). Moreover, they release CCN1, a matricellular protein that will induce senescence in surrounding cells to prevent fibrosis (Jun and Lau, 2010). Senescent cells initiate immune responses by secreting pro-inflammatory factors such as IL-6 at the site of the wound, where immune cells then clear senescent cells. IL-6 has also been shown to increase cellular plasticity to promote tissue regeneration (Andrade et al., 2022). Despite their beneficial roles in processes such as embryonic development, wound healing, and tumor suppression, accumulation of these cells can cause chronic inflammation (Andrade et al., 2022). To maintain this homeostasis, senescent cells utilize SASP factors to recruit cells from the immune system, which initiate a response to clear senescent cells before they amass (Majewska and Krizhanovsky, 2025b). The immune system is generally divided into two types of responses: the innate immune system and the adaptive immune system (Wang et al., 2020). Each response features immune cell types that utilize different mechanisms to clear senescent cells or communicate with other immune cells for further response (Figure 2).

Innate cells are a group of immune cells that act as the body’s first line of defense. Due to its non-specificity, they recognize a vast number of pathogens (Wang et al., 2024). Such cells include phagocytes (neutrophils and macrophages), lymphocytes like natural killer cells (NK), and granulocytes such as eosinophils and dendritic cells (DC) (Wang et al., 2024). Innate immune cells recognize pathogens via pattern-associated molecular patterns (PAMPs)-conserved regions within pathogens, or by damage-associated molecular patterns (DAMPs). Using pattern recognition receptors (PRRs), innate cells bind to PAMPs or DAMPs and initiate responses to effectively eliminate pathogens (Wang et al., 2024). When a senescent cell is detected by the innate immune system, these cells serve distinct roles in the removal of senescent cells or recruitment of immune cells to facilitate clearance. Myeloid cells, including monocytes and macrophages, are key innate immune cell types that regulate senescent cell burden (Prata et al., 2018). In the context of normal physiological conditions, the SASP released by senescent cells acts as a chemotactic gradient to recruit macrophages facilitate senescent cell clearance (Deng et al., 2026). Senescent monocytes have also been demonstrated to have a pro-inflammatory and enhanced ability to adhere to endothelial cells and possibly promote vascular disease (Merino et al., 2011; Olinger et al., 2025). Specifically, acute senescent cells upregulate p21^CIP1^ expression through the p53/p21^CIP1^ pathway, which rapidly gives rise to chemokines such as CXCL14, which in turn recruits macrophages towards senescent cells (Sturmlechner et al., 2021). Notably, macrophages do not immediately attack the cell; instead, they reside near the senescent cell and monitor it determine an outcome. If p21^CIP1^ expression persists, macrophages polarize into their pro-inflammatory (M1) state and undergo phagocytosis (engulf and digest) to clear senescent cells (Sturmlechner et al., 2021). Macrophages will also release pro-inflammatory cytokines (e.g., TNF-α, IL-6) to further recruit T cells and other immune cell types (Majewska and Krizhanovsky, 2025b). NK cells are activated via the NKG2D receptor that recognizes ligands on the surface of senescent cells (Sagiv et al., 2016). Upon activation, NK cells release cytotoxic granules, which contain perforin and granzymes, to induce apoptosis (Lanier, 2024). They will also express IFN-γ to activate other immune cells (Gergues et al., 2025). Neutrophils can be recruited by SASP factors such as IL-1β and TNF-α and will contribute to the clearance of senescent cells by releasing Neutrophil Extracellular Traps (NETs)- webs of DNA with granule proteins (Binet et al., 2020). Activated neutrophils can also utilize phagocytosis to clear senescent cells; however, this role is more prominent in macrophages. Neutrophils will also recruit other immune cells for clearance as well. Recent studies have suggested that eosinophils may aid in reducing low-grade inflammation. One study found that when young eosinophils were administered into aged mice, white adipose tissue (WAT) in aged mice displayed reduced inflammation (Brigger et al., 2020); however, research in this area is sparse. Dendritic cells (DC) are antigen-presenting cells that activate T cells by presenting senescence-related antigens, acting as a bridge from innate immune cells to the adaptive immune cells (Majewska and Krizhanovsky, 2025b).

While innate immune cells can rapidly respond to pathogenic challenges, adaptive immunity requires several days to generate highly-specific immune responses (Warrick et al., 2025). Because of its highly specific nature, adaptive immune responses develop memory to recognize pathogens to rapidly respond to future re-infections. The adaptive immune system is composed of B and T lymphocytes, which mature and differentiate into various types; B cells can become memory B cells and plasma cells, whereas T cells can differentiate into helper cells (CD4^+^), cytotoxic (CD8^+^), regulatory (Tregs), and memory T cells (Wang et al., 2024). Research suggests that senescent cells can upregulate MHC-I antigen processing machinery, making them highly sensitive to CD8^+^ T cell-mediated elimination (Marin et al., 2023). Senescent cells can release senescence-related antigens (seno-antigens) as well, resulting in DC activation and antigen-specific T cell responses (Marin et al., 2023). CD8^+^ T cells will eliminate senescent cells by releasing granules and perforins, similarly to NK cells who will also induce apoptosis via Fas/FasL pathway (Voskoboinik et al., 2015; Ovadya et al., 2018; Marin et al., 2023; Wang et al., 2024). CD4^+^ T cells act as support in the clearance of senescent cells by producing cytokines such as IL-2 to maintain the proliferation of CD8^+^ T cells (Niederlova et al., 2023). CD4^+^ T cells can become cytotoxic (CD4^+^ CTL) as well when cross-presented with MHC-II and recently with HLA-E, particularly in senescent human fibroblasts (Hasegawa et al., 2023). Depending on the senescent cell burden, the transcription factor Eomesodermin (EOMES) can also induce differentiation of CD4^+^ T cells into a cytotoxic variant (Elyahu et al., 2025). Tregs have been shown to mediate homeostasis within the immune system by balancing the immune response from effector cells (Sakaguchi et al., 2008). More recently, B cell vaccines that target seno-antigens like GPNMB are being researched as a potential therapeutic to target senescent cells (Suda et al., 2021). However, due to the lack of a distinct universal marker of senescence, such therapeutics remain a challenge (Ogrodnik et al., 2024).

## Mechanisms of immune evasion by senescent cells

While acute senescent cells are typically cleared by the immune system, chronic senescent cells have developed mechanisms to evade surveillance, thereby allowing these cells to accumulate within tissues (Figure 2). Senescent cells can upregulate CD47/SIRPα, commonly known as the “do not eat me signal” (Kojima et al., 2016). Normally, dying cells lose CD47 expression over time, which triggers macrophages, neutrophils, and DC cells to clear them. Because senescent cells continue to express CD47, it binds to the SIRPα receptors on these cells, shielding them from clearance (Majewska and Krizhanovsky, 2025b). In human fibroblasts and endothelial cells, it was shown that senescent cells increase expression of HLE-A, which binds to the NKG2A receptor on NK cells, thereby inhibiting their response (Pereira et al., 2019). Senescent cells can shed NKG2D ligands (e.g., MICA, ULBP2) via matrix metalloproteinase (MMP) mediated cleavage, evading NK cell surveillance (Zingoni et al., 2015). A recent study suggests that mice with liver or lung fibrosis harbor senescent cells that also express GD3 ganglioside, which may also inhibit NK cell function (Majewska and Krizhanovsky, 2025a). T cell responses can be reduced by upregulating PD-L1, which binds to PD-1 on CD8^+^ and CD4^+^ T cells, inhibiting their cytotoxic abilities (Wang et al., 2022; Majewska et al., 2024). Studies have also shown that tumor-associated macrophages (TAMs) that express PD-1 have impaired functionality as well (Gordon et al., 2017). Tregs may also contribute to immune escape of senescent cells by suppressing cytotoxic T cells (Rocamora-Reverte et al., 2020). Senescent cells can secrete SASP factors IL-10 and TGF-β, which drive the expansion of myeloid-derived suppressor cells (MDSCs), which in turn expand other immunosuppressive cells such as M2 macrophages (Salminen, 2024). Recently, it was found that senescent fibroblasts may have increased Fas ligand (FasL) expression which not only recruits immune cells but also promotes apoptosis of T and NK cells (Cruz-Barrera et al., 2025). Furthermore, SASP factors secreted by senescent cells, along with recruitment of immunosuppressive cells and upregulation of inhibitory pathways, lead to exhausted immune cells, allowing senescent cells to evade clearance (Wang et al., 2024). Intriguingly, it was discovered that in senescent mesenchymal stem cells (MSCs), expression of extracellular matrix (ECM) proteins and enzymes increased, leading to altered composition of tissue environments seen in breast cancer (Ghosh et al., 2020). Though more research is needed to better understand the impact of senescence-related changes in the ECM on immunosurveillance.

## Immunosenescence impairs immunosurveillance

Not only do senescent cells evolve mechanisms to escape immune clearance, but with age changes in immune cell populations, both in numbers and function, causes an intrinsic decline in immune surveillance. Immunosenescence, a term introduced by Roy Walford 6 decades ago, describes the gradual, age-related reshaping and weakening of immune function (Walford, 1964). As it progresses, older individuals show poorer protection against infections, lower responses to vaccination, and a greater likelihood of developing diseases associated with aging (Pawelec et al., 2020). Immune senescence is driven by multiple events including thymic involution, a shift of hematopoietic stem cells (HSCs) toward myeloid lineages that can impair lymphopoiesis, loss of naïve cells and other shifts in populations, and impair responses to stimuli and the production of a chronic inflammatory milieu (Liu et al., 2023). These alterations reflect disturbances in both innate and adaptive immunity and is linked to thymic involution, persistent low-grade inflammation (“inflammaging”), and the build-up of senescent cells (Wrona et al., 2024; Franceschi et al., 2025). Senescent cells and the SASP they produce are key contributors to inflammaging (De Luca et al., 2025; Ghosh et al., 2026). Senescent cells can recruit inflammatory monocytes via CCL2 production, which can cause localized tissue damage through aberrant release of pro-inflammatory molecules (Albright et al., 2016). Infiltrating monocytes from older individuals have been shown to produce elevated levels of lipid mediators, like prostaglandin E2, which can inhibit downstream T cell activation and proliferation and impair antigen-specific tissue responses (Chambers et al., 2021). Furthermore, the development of senescence in different immune cell types has been reported (Chen et al., 2009; Martinez-Zamudio et al., 2021; Yousefzadeh et al., 2021; Turano et al., 2025; Salladay-Perez et al., 2026).

Immune cells of multiple fates (e.g., exhausted, senescent, or otherwise dysfunctional states) combine to not only impair immune surveillance, but immune cells can adopt a hyperinflammatory state (Delgado-Pulido et al., 2025). When immunosurveillance falters, especially with age, senescent cells are able to not only endure but also to accumulate. In this setting, immune cells may cluster around senescent cells without resolving the process, promoting persistent inflammation and immune exhaustion (Gil, 2025). Failed surveillance can also worsen disease contexts: in tumors, it supports a pro-tumor environment, and in organs such as the liver, lungs, and kidneys, it promotes fibrosis and functional decline (Coppe et al., 2010). Senescent cells further reinforce dysfunction by releasing immunosuppressive factors, altering NK-cell signaling via surface ligands, and impairing dendritic cell maturation and antigen presentation—creating a self-amplifying cycle that can accelerate immunosenescence (Gil, 2025). Studies demonstrating exploring the role of genomic instability in hematopoietic cells or mitochondrial dysfunction specifically in T cells in immunosenescence highlight how premature immune aging fosters the unrestrained propagation of senescent cell burden in the parenchyma, ultimately shortening both healthspan and lifespan (Desdin-Mico et al., 2020; Yousefzadeh et al., 2021; Jang et al., 2026).

Due to the impact of immunosenescence on immune and systemic aging, considerable focus is placed on restoring immune function lost to age (Wrona et al., 2024). Thymic function can be boosted by restoring thymopoiesis via exogenous RANKL or FOXN1 gene therapy to revitalize the thymic microenvironment (Santamaria et al., 2024). At the same time, HSC aging may be counteracted by mTOR or p38 MAPK inhibition or antibody-mediated depletion of CD150^+^ myeloid-skewed HSCs (Chen et al., 2009; Sorimachi et al., 2021; Ross et al., 2024). Epigenetically fixed exhaustion in CD8^+^ T cells may be relieved with demethylating drugs (e.g., decitabine); and cytotoxic antiviral/antitumor activity in older CD8^+^ T cells can be strengthened via PD-1 blockade (Li et al., 2025). Lastly, a recent study has demonstrated that targeting key transcription factor networks (RUNX2, KLF5, and cJUN) can partially restore deficits in TCR-mediated stimulation present in senescent CD8^+^ T cells (Turano et al., 2026).

The use of senolytics and other gerotherapeutics like interventions have had positive effects on immune function in older humans and mice (Wrona et al., 2024). Because NK function declines with age, strategies such as NKG2D-specific chimeric antigen receptor (CAR)-T cells and transfer of expanded autologous NK cells have shown promise in lowering senescence and inflammatory markers in preclinical studies and early human work (Nakazawa et al., 2025). Senescent cells can also evade immunity by upregulating PD-L1, and PD-L1 blockade can further reduce senescent cell burden in disease models (Majewska et al., 2024). Furthermore, recent work has shown that gerotherapeutics like the sodium-glucose co-transporter two inhibitor canagliflozen can stimulate immunosurveillance by reducing expression of PD-L1 on the surface of senescent cells (Katsuumi et al., 2024). More targeted “search-and-destroy” platforms are also emerging, particularly uPAR-directed CAR-T cells, which can clear senescent cells and improve metabolic and fibrotic phenotypes in aging models (Amor et al., 2020; Amor et al., 2024; Rosas-Campos et al., 2025). Related uPAR-specific CAR-macrophages (CAR-M) may add benefits by combining senescent-cell engulfment with extracellular-matrix remodeling and recruitment of endogenous T cells, offering potential utility in fibrotic disorders such as liver fibrosis (Dai et al., 2024).

## Conclusion

Senescent cells represent a paradox in biology; they are an essential component in normal physiological processes like wound healing, tumor supression and embryonic development (de Magalhaes, 2024). However, as we age, our immune system quality declines; resulting in these cells accumulating and contributing to the onset of age-related diseases (Jang et al., 2026). Considerable focus in the literature is given to their formation and accumulation with aging and disease or exploiting intrinsic pathways such as senescent cell anti-apoptotic pathways or other molecular alterations that are commonly targeted by senotherapeutics (Zhang et al., 2022a; Zhang et al., 2022b). However, the role of the immune system in responding to and constraining senescent cell burden should be appreciated and is of particular importance (Majewska and Krizhanovsky, 2025b). The role of the immune system protecting against or (in some contexts) driving systemic aging has become appreciated (Jang et al., 2026). Many approaches are being investigated to rejuvenate an aged immune system but it remains to be seen whether these interventions are enhancing immunosurveillance of senescent cells (either directly or indirectly) or whether their benefits are derived through other mechanisms such as restoring immune resolution or ameliorating the pathogenic features that accumulate in some aged immune cells (Wrona et al., 2024; Majewska and Krizhanovsky, 2025b). Improvements in correcting age-related deficits in immune surveillance should not only reduce senescent cell burden but also promote healthy aging through the removal of dysfunctional or neoplastic cells that can accumulate with age.

## References

[B1] Acosta J. C. Banito A. Wuestefeld T. Georgilis A. Janich P. Morton J. P. (2013). A complex secretory program orchestrated by the inflammasome controls paracrine senescence. Nat. Cell. Biol. 15 (8), 978–990. 10.1038/ncb2784 23770676 PMC3732483

[B2] Albright J. M. Dunn R. C. Shults J. A. Boe D. M. Afshar M. Kovacs E. J. (2016). Advanced age alters monocyte and macrophage responses. Antioxid. Redox Signal 25 (15), 805–815. 10.1089/ars.2016.6691 27357201 PMC5107740

[B3] Amor C. Feucht J. Leibold J. Ho Y. J. Zhu C. Alonso-Curbelo D. (2020). Senolytic CAR T cells reverse senescence-associated pathologies. Nature 583 (7814), 127–132. 10.1038/s41586-020-2403-9 32555459 PMC7583560

[B4] Amor C. Fernandez-Maestre I. Chowdhury S. Ho Y. J. Nadella S. Graham C. (2024). Prophylactic and long-lasting efficacy of senolytic CAR T cells against age-related metabolic dysfunction. Nat. Aging 4 (3), 336–349. 10.1038/s43587-023-00560-5 38267706 PMC10950785

[B5] Andrade A. M. Sun M. Gasek N. S. Hargis G. R. Sharafieh R. Xu M. (2022). Role of senescent cells in cutaneous wound healing. Biol. (Basel) 11 (12), 1731. 10.3390/biology11121731 PMC9775319 36552241

[B6] Binet F. Cagnone G. Crespo-Garcia S. Hata M. Neault M. Dejda A. (2020). Neutrophil extracellular traps target senescent vasculature for tissue remodeling in retinopathy. Science 369 (6506). 10.1126/science.aay5356 32820093

[B7] Borgoni S. Kudryashova K. S. Burka K. de Magalhaes J. P. (2021). Targeting immune dysfunction in aging. Ageing Res. Rev. 70, 101410. 10.1016/j.arr.2021.101410 34280555

[B8] Brigger D. Riether C. van Brummelen R. Mosher K. I. Shiu A. Ding Z. (2020). Eosinophils regulate adipose tissue inflammation and sustain physical and immunological fitness in old age. Nat. Metab. 2 (8), 688–702. 10.1038/s42255-020-0228-3 32694825 PMC7438316

[B9] Chambers E. S. Vukmanovic-Stejic M. Shih B. B. Trahair H. Subramanian P. Devine O. P. (2021). Recruitment of inflammatory monocytes by senescent fibroblasts inhibits antigen-specific tissue immunity during human aging. Nat. Aging 1 (1), 101–113. 10.1038/s43587-020-00010-6 37118005

[B10] Chen C. Liu Y. Liu Y. Zheng P. (2009). mTOR regulation and therapeutic rejuvenation of aging hematopoietic stem cells. Sci. Signal 2 (98), ra75. 10.1126/scisignal.2000559 19934433 PMC4020596

[B11] Chen H. A. Ho Y. J. Mezzadra R. Adrover J. M. Smolkin R. Zhu C. (2023). Senescence rewires microenvironment sensing to facilitate antitumor immunity. Cancer Discov. 13 (2), 432–453. 10.1158/2159-8290.CD-22-0528 36302222 PMC9901536

[B12] Coppe J. P. Desprez P. Y. Krtolica A. Campisi J. (2010). The senescence-associated secretory phenotype: the dark side of tumor suppression. Annu. Rev. Pathol. 5, 99–118. 10.1146/annurev-pathol-121808-102144 20078217 PMC4166495

[B13] Cruz-Barrera M. Dulong J. Mansour Nehmo G. Sonn A. Moquin-Beaudry G. Benabdallah B. (2025). Senescent human fibroblasts have increased FasL expression and impair the tumor immune response. Front. Immunol. 16, 1685269. 10.3389/fimmu.2025.1685269 41221280 PMC12597918

[B14] Dai H. Zhu C. Huai Q. Xu W. Zhu J. Zhang X. (2024). Chimeric antigen receptor-modified macrophages ameliorate liver fibrosis in preclinical models. J. Hepatol. 80 (6), 913–927. 10.1016/j.jhep.2024.01.034 38340812

[B15] De Luca F. Camporeale V. Leccese G. Cuttano R. Troise D. Infante B. (2025). From senescent cells to systemic inflammation: the role of inflammaging in age-related diseases and kidney dysfunction. Cells 14 (22), 1831. 10.3390/cells14221831 41294884 PMC12651686

[B16] de Magalhaes J. P. (2024). Cellular senescence in normal physiology. Science 384 (6702), 1300–1301. 10.1126/science.adj7050 38900869

[B17] Delgado-Pulido S. Yousefzadeh M. J. Mittelbrunn M. (2025). Aging reshapes the adaptive immune system from healer to saboteur. Nat. Aging 5 (8), 1393–1403. 10.1038/s43587-025-00906-1 40813808

[B18] Demaria M. Ohtani N. Youssef S. A. Rodier F. Toussaint W. Mitchell J. R. (2014). An essential role for senescent cells in optimal wound healing through secretion of PDGF-AA. Dev. Cell. 31 (6), 722–733. 10.1016/j.devcel.2014.11.012 25499914 PMC4349629

[B19] Deng X. Yin Z. Tai S. Wang Y. Fu L. (2026). Macrophage senescence: Friend or foe? Aging Dis. 369. 10.14336/AD.2025.1394 41701874

[B20] Desdin-Mico G. Soto-Heredero G. Aranda J. F. Oller J. Carrasco E. Gabande-Rodriguez E. (2020). T cells with dysfunctional mitochondria induce multimorbidity and premature senescence. Science 368 (6497), 1371–1376. 10.1126/science.aax0860 32439659 PMC7616968

[B21] Dimri G. P. Lee X. Basile G. Acosta M. Scott G. Roskelley C. (1995). A biomarker that identifies senescent human cells in culture and in aging skin *in vivo* . Proc. Natl. Acad. Sci. U. S. A. 92 (20), 9363–9367. 10.1073/pnas.92.20.9363 7568133 PMC40985

[B22] Dou Z. Ghosh K. Vizioli M. G. Zhu J. Sen P. Wangensteen K. J. (2017). Cytoplasmic chromatin triggers inflammation in senescence and cancer. Nature 550 (7676), 402–406. 10.1038/nature24050 28976970 PMC5850938

[B23] Elyahu Y. Feygin I. Eremenko E. Pinkas N. Zemer A. Shicht A. (2025). CD4 T cells acquire eomesodermin to modulate cellular senescence and aging. Nat. Aging 5, 1970–1982. 10.1038/s43587-025-00953-8 41057611

[B24] Feng T. Xie F. Lee L. M. Y. Lin Z. Tu Y. Lyu Y. (2025). Cellular senescence in cancer: from mechanism paradoxes to precision therapeutics. Mol. Cancer 24 (1), 213. 10.1186/s12943-025-02419-2 40781676 PMC12333312

[B25] Franceschi C. Bonafe M. Valensin S. Olivieri F. De Luca M. Ottaviani E. (2000). Inflamm-aging. An evolutionary perspective on immunosenescence. Ann. N. Y. Acad. Sci. 908, 244–254. 10.1111/j.1749-6632.2000.tb06651.x 10911963

[B26] Franceschi C. Olivieri F. Moskalev A. Ivanchenko M. Santoro A. (2025). Toward precision interventions and metrics of inflammaging. Nat. Aging 5 (8), 1441–1454. 10.1038/s43587-025-00938-7 40813813

[B27] Freund A. Laberge R. M. Demaria M. Campisi J. (2012). Lamin B1 loss is a senescence-associated biomarker. Mol. Biol. Cell. 23 (11), 2066–2075. 10.1091/mbc.E11-10-0884 22496421 PMC3364172

[B28] Fumagalli M. Rossiello F. Mondello C. d'Adda di Fagagna F. (2014). Stable cellular senescence is associated with persistent DDR activation. PLoS One 9 (10), e110969. 10.1371/journal.pone.0110969 25340529 PMC4207795

[B29] Gergues M. Bari R. Koppisetti S. Gosiewska A. Kang L. Hariri R. J. (2025). Senescence, NK cells, and cancer: navigating the crossroads of aging and disease. Front. Immunol. 16, 1565278. 10.3389/fimmu.2025.1565278 40255394 PMC12006071

[B30] Ghosh D. Mejia Pena C. Quach N. Xuan B. Lee A. H. Dawson M. R. (2020). Senescent mesenchymal stem cells remodel extracellular matrix driving breast cancer cells to a more-invasive phenotype. J. Cell. Sci. 133 (2). 10.1242/jcs.232470 31932504 PMC6983709

[B31] Ghosh R. Dipali S. S. Desdin-Mico G. Yousefzadeh M. J. (2026). Communication breakdown: senescent cells in interorgan communication of aging. Trends Endocrinol. Metab. 10.1016/j.tem.2026.02.002 PMC12991428 41813553

[B32] Gil J. (2025). The interplay between senescence, inflammation, and the immune system. Genes. Dev. 39 (15-16), 923–925. 10.1101/gad.353125.125 40645668 PMC12315857

[B33] Gordon S. R. Maute R. L. Dulken B. W. Hutter G. George B. M. McCracken M. N. (2017). PD-1 expression by tumour-associated macrophages inhibits phagocytosis and tumour immunity. Nature 545 (7655), 495–499. 10.1038/nature22396 28514441 PMC5931375

[B34] Hasegawa T. Oka T. Son H. G. Oliver-Garcia V. S. Azin M. Eisenhaure T. M. (2023). Cytotoxic CD4(+) T cells eliminate senescent cells by targeting cytomegalovirus antigen. Cell. 186 (7), 1417–1431 e1420. 10.1016/j.cell.2023.02.033 37001502

[B35] Huang W. Hickson L. J. Eirin A. Kirkland J. L. Lerman L. O. (2022). Cellular senescence: the good, the bad and the unknown. Nat. Rev. Nephrol. 18 (10), 611–627. 10.1038/s41581-022-00601-z 35922662 PMC9362342

[B36] Jackstadt R. Jung P. Hermeking H. (2013). AP4 directly downregulates p16 and p21 to suppress senescence and mediate transformation. Cell. Death Dis. 4 (8), e775. 10.1038/cddis.2013.282 23949224 PMC3763444

[B37] Jang I. H. Niedernhofer L. J. Robbins P. D. Camell C. D. (2026). The ageing immune system as a driver of systemic ageing. Nat. Rev. Immunol. 26, 489–506. 10.1038/s41577-026-01269-3 41688787 PMC13723950

[B38] Jun J. I. Lau L. F. (2010). The matricellular protein CCN1 induces fibroblast senescence and restricts fibrosis in cutaneous wound healing. Nat. Cell. Biol. 12 (7), 676–685. 10.1038/ncb2070 20526329 PMC2919364

[B39] Katsuumi G. Shimizu I. Suda M. Yoshida Y. Furihata T. Joki Y. (2024). SGLT2 inhibition eliminates senescent cells and alleviates pathological aging. Nat. Aging 4 (7), 926–938. 10.1038/s43587-024-00642-y 38816549 PMC11257941

[B40] Khosla S. Monroe D. G. Farr J. N. (2025). Towards a personalized approach in senolytic trials. Nat. Aging 5 (10), 1926–1929. 10.1038/s43587-025-00964-5 40926125

[B41] Kojima Y. Volkmer J. P. McKenna K. Civelek M. Lusis A. J. Miller C. L. (2016). CD47-blocking antibodies restore phagocytosis and prevent atherosclerosis. Nature 536 (7614), 86–90. 10.1038/nature18935 27437576 PMC4980260

[B42] Lanier L. L. (2024). Five decades of natural killer cell discovery. J. Exp. Med. 221 (8), e20231222. 10.1084/jem.20231222 38842526 PMC11157086

[B43] Li C. Yuan Y. Jiang X. Wang Q. (2025). Epigenetic regulation of CD8(+) T cell exhaustion: recent advances and update. Front. Immunol. 16, 1700039. 10.3389/fimmu.2025.1700039 41194917 PMC12582961

[B44] Liu Z. Liang Q. Ren Y. Guo C. Ge X. Wang L. (2023). Immunosenescence: molecular mechanisms and diseases. Signal Transduct. Target Ther. 8 (1), 200. 10.1038/s41392-023-01451-2 37179335 PMC10182360

[B45] Lopez-Otin C. Blasco M. A. Partridge L. Serrano M. Kroemer G. (2013). The hallmarks of aging. Cell. 153 (6), 1194–1217. 10.1016/j.cell.2013.05.039 23746838 PMC3836174

[B46] Lopez-Otin C. Blasco M. A. Partridge L. Serrano M. Kroemer G. (2023). Hallmarks of aging: an expanding universe. Cell. 186 (2), 243–278. 10.1016/j.cell.2022.11.001 36599349

[B47] Majewska J. Krizhanovsky V. (2025a). GD3 ganglioside checkpoints in immune surveillance of senescent cells. Nat. Aging 5 (2), 182–183. 10.1038/s43587-025-00803-7 39814961

[B48] Majewska J. Krizhanovsky V. (2025b). Immune surveillance of senescent cells in aging and disease. Nat. Aging 5 (8), 1415–1424. 10.1038/s43587-025-00910-5 40813810

[B49] Majewska J. Agrawal A. Mayo A. Roitman L. Chatterjee R. Sekeresova Kralova J. (2024). p16-dependent increase of PD-L1 stability regulates immunosurveillance of senescent cells. Nat. Cell. Biol. 26 (8), 1336–1345. 10.1038/s41556-024-01465-0 39103548 PMC11321988

[B50] Marin I. Serrano M. Pietrocola F. (2023). Recent insights into the crosstalk between senescent cells and CD8 T lymphocytes. NPJ Aging 9 (1), 8. 10.1038/s41514-023-00105-5 37015935 PMC10073090

[B51] Martinez-Zamudio R. I. Roux P. F. de Freitas J. Robinson L. Dore G. Sun B. (2020). AP-1 imprints a reversible transcriptional programme of senescent cells. Nat. Cell. Biol. 22 (7), 842–855. 10.1038/s41556-020-0529-5 32514071 PMC7899185

[B52] Martinez-Zamudio R. I. Dewald H. K. Vasilopoulos T. Gittens-Williams L. Fitzgerald-Bocarsly P. Herbig U. (2021). Senescence-associated beta-galactosidase reveals the abundance of senescent CD8+ T cells in aging humans. Aging Cell. 20 (5), e13344. 10.1111/acel.13344 33939265 PMC8135084

[B53] Martinez-Zamudio R. I. Stefa A. Nabuco Leva Ferreira Freitas J. A. Vasilopoulos T. Simpson M. Dore G. (2023). Escape from oncogene-induced senescence is controlled by POU2F2 and memorized by chromatin scars. Cell. Genom 3 (4), 100293. 10.1016/j.xgen.2023.100293 37082139 PMC10112333

[B54] Merino A. Buendia P. Martin-Malo A. Aljama P. Ramirez R. Carracedo J. (2011). Senescent CD14+CD16+ monocytes exhibit proinflammatory and proatherosclerotic activity. J. Immunol. 186 (3), 1809–1815. 10.4049/jimmunol.1001866 21191073

[B55] Miller K. N. Victorelli S. G. Salmonowicz H. Dasgupta N. Liu T. Passos J. F. (2021). Cytoplasmic DNA: sources, sensing, and role in aging and disease. Cell. 184 (22), 5506–5526. 10.1016/j.cell.2021.09.034 34715021 PMC8627867

[B56] Nakazawa T. Yamanishi R. Morimoto T. Matusda R. (2025). Natural killer cell-based senotherapy: a promising strategy for healthy aging. Front. Immunol. 16, 1737572. 10.3389/fimmu.2025.1737572 41601660 PMC12833471

[B57] Niederlova V. Tsyklauri O. Kovar M. Stepanek O. (2023). IL-2-driven CD8(+) T cell phenotypes: implications for immunotherapy. Trends Immunol. 44 (11), 890–901. 10.1016/j.it.2023.09.003 37827864 PMC7615502

[B58] Ogrodnik M. Carlos Acosta J. Adams P. D. d'Adda di Fagagna F. Baker D. J. Bishop C. L. (2024). Guidelines for minimal information on cellular senescence experimentation *in vivo* . Cell. 187 (16), 4150–4175. 10.1016/j.cell.2024.05.059 39121846 PMC11790242

[B59] Olinger B. Banarjee R. Dey A. Tsitsipatis D. Tanaka T. Ram A. (2025). The secretome of senescent monocytes predicts age-related clinical outcomes in humans. Nat. Aging 5 (7), 1266–1279. 10.1038/s43587-025-00877-3 40461807 PMC12276915

[B60] Ovadya Y. Landsberger T. Leins H. Vadai E. Gal H. Biran A. (2018). Impaired immune surveillance accelerates accumulation of senescent cells and aging. Nat. Commun. 9 (1), 5435. 10.1038/s41467-018-07825-3 30575733 PMC6303397

[B61] Pawelec G. Bronikowski A. Cunnane S. C. Ferrucci L. Franceschi C. Fulop T. (2020). The conundrum of human immune system senescence. Mech. Ageing Dev. 192, 111357. 10.1016/j.mad.2020.111357 32949594 PMC7494491

[B62] Pereira B. I. Devine O. P. Vukmanovic-Stejic M. Chambers E. S. Subramanian P. Patel N. (2019). Senescent cells evade immune clearance *via* HLA-E-mediated NK and CD8(+) T cell inhibition. Nat. Commun. 10 (1), 2387. 10.1038/s41467-019-10335-5 31160572 PMC6547655

[B63] Prata L. Ovsyannikova I. G. Tchkonia T. Kirkland J. L. (2018). Senescent cell clearance by the immune system: emerging therapeutic opportunities. Semin. Immunol. 40, 101275. 10.1016/j.smim.2019.04.003 31088710 PMC7061456

[B64] Reimann M. Lee S. Schmitt C. A. (2024). Cellular senescence: neither irreversible nor reversible. J. Exp. Med. 221 (4), e20232136. 10.1084/jem.20232136 38385946 PMC10883852

[B65] Rocamora-Reverte L. Melzer F. L. Wurzner R. Weinberger B. (2020). The complex role of regulatory T cells in immunity and aging. Front. Immunol. 11, 616949. 10.3389/fimmu.2020.616949 33584708 PMC7873351

[B66] Roger L. Tomas F. Gire V. (2021). Mechanisms and regulation of cellular senescence. Int. J. Mol. Sci. 22 (23), 13173. 10.3390/ijms222313173 34884978 PMC8658264

[B67] Rosas-Campos R. Arceo-Orozco S. Sandoval-Rodriguez A. Madrigal J. A. Armendariz-Borunda J. (2025). Above and beyond senescence and CAR T cell: advances and future perspectives. Front. Immunol. 16, 1701655. 10.3389/fimmu.2025.1701655 41476987 PMC12747912

[B68] Ross J. B. Myers L. M. Noh J. J. Collins M. M. Carmody A. B. Messer R. J. (2024). Depleting myeloid-biased haematopoietic stem cells rejuvenates aged immunity. Nature 628 (8006), 162–170. 10.1038/s41586-024-07238-x 38538791 PMC11870232

[B69] Sagiv A. Burton D. G. Moshayev Z. Vadai E. Wensveen F. Ben-Dor S. (2016). NKG2D ligands mediate immunosurveillance of senescent cells. Aging (Albany NY) 8 (2), 328–344. 10.18632/aging.100897 26878797 PMC4789586

[B70] Sakaguchi S. Yamaguchi T. Nomura T. Ono M. (2008). Regulatory T cells and immune tolerance. Cell. 133 (5), 775–787. 10.1016/j.cell.2008.05.009 18510923

[B71] Salladay-Perez I. A. Avila I. Estrada L. Alexandru A. C. Ponce C. Dhingra A. (2026). p21(+)TREM2(+) senescent macrophages fuel inflammaging and metabolic dysfunction-associated steatotic liver disease. Nat. Aging 6 (4), 792–815. 10.1038/s43587-026-01101-6 41991686 PMC13099426

[B72] Salminen A. (2024). Inhibitory immune checkpoints suppress the surveillance of senescent cells promoting their accumulation with aging and in age-related diseases. Biogerontology 25 (5), 749–773. 10.1007/s10522-024-10114-w 38954358 PMC11374851

[B73] Santamaria J. C. Chevallier J. Dutour L. Picart A. Kergaravat C. Cieslak A. (2024). RANKL treatment restores thymic function and improves T cell-mediated immune responses in aged mice. Sci. Transl. Med. 16 (776), eadp3171. 10.1126/scitranslmed.adp3171 39630886

[B74] Sharpless N. E. Sherr C. J. (2015). Forging a signature of *in vivo* senescence. Nat. Rev. Cancer 15 (7), 397–408. 10.1038/nrc3960 26105537

[B75] Soriani A. Zingoni A. Cerboni C. Iannitto M. L. Ricciardi M. R. Di Gialleonardo V. (2009). ATM-ATR-dependent up-regulation of DNAM-1 and NKG2D ligands on multiple myeloma cells by therapeutic agents results in enhanced NK-cell susceptibility and is associated with a senescent phenotype. Blood 113 (15), 3503–3511. 10.1182/blood-2008-08-173914 19098271

[B76] Soriani A. Fionda C. Ricci B. Iannitto M. L. Cippitelli M. Santoni A. (2013). Chemotherapy-elicited upregulation of NKG2D and DNAM-1 ligands as a therapeutic target in multiple myeloma. Oncoimmunology 2 (12), e26663. 10.4161/onci.26663 24498552 PMC3912005

[B77] Sorimachi Y. Karigane D. Ootomo Y. Kobayashi H. Morikawa T. Otsu K. (2021). p38alpha plays differential roles in hematopoietic stem cell activity dependent on aging contexts. J. Biol. Chem. 296, 100563. 10.1016/j.jbc.2021.100563 33745970 PMC8065231

[B78] Sturmlechner I. Zhang C. Sine C. C. van Deursen E. J. Jeganathan K. B. Hamada N. (2021). p21 produces a bioactive secretome that places stressed cells under immunosurveillance. Science 374 (6567), eabb3420. 10.1126/science.abb3420 34709885 PMC8985214

[B79] Suda M. Shimizu I. Katsuumi G. Yoshida Y. Hayashi Y. Ikegami R. (2021). Senolytic vaccination improves normal and pathological age-related phenotypes and increases lifespan in progeroid mice. Nat. Aging 1 (12), 1117–1126. 10.1038/s43587-021-00151-2 37117524

[B80] Turano P. S. Akbulut E. Aquino N. M. Garza-Martinez L. Singh S. Yap G. S. (2025). “Senescent CD8 T effector memory cells are functionally impaired,” in Enriched in Aging and Disease, and a Barrier to Immunotherapy. bioRxiv. 10.64898/2025.12.16.694716

[B81] Turano P. S. Akbulut E. Dewald H. K. Vasilopoulos T. Fitzgerald-Bocarsly P. Herbig U. (2026). Age-independent and targetable transcription factor networks regulating CD8(+) T cell senescence in aging humans. Cell. Rep. 45 (1), 116795. 10.1016/j.celrep.2025.116795 41481417 PMC12856879

[B82] Voskoboinik I. Whisstock J. C. Trapani J. A. (2015). Perforin and granzymes: function, dysfunction and human pathology. Nat. Rev. Immunol. 15 (6), 388–400. 10.1038/nri3839 25998963

[B83] Walford R. L. (1964). The immunologic theory of aging. Gerontologist 4, 195–197. 10.1093/geront/4.4.195 14289265

[B84] Wang T. W. Nakanishi M. (2025). Immune surveillance of senescence: potential application to age-related diseases. Trends Cell. Biol. 35 (3), 248–257. 10.1016/j.tcb.2024.06.007 39025762

[B85] Wang R. Lan C. Benlagha K. Camara N. O. S. Miller H. Kubo M. (2020). The interaction of innate immune and adaptive immune system. MedComm 5 (10), e714. 10.1002/mco2.714 PMC11401974 39286776

[B86] Wang T. W. Johmura Y. Suzuki N. Omori S. Migita T. Yamaguchi K. (2022). Blocking PD-L1-PD-1 improves senescence surveillance and ageing phenotypes. Nature 611 (7935), 358–364. 10.1038/s41586-022-05388-4 36323784

[B87] Wang B. Han J. Elisseeff J. H. Demaria M. (2024). The senescence-associated secretory phenotype and its physiological and pathological implications. Nat. Rev. Mol. Cell. Biol. 25 (12), 958–978. 10.1038/s41580-024-00727-x 38654098

[B88] Warrick K. A. Vallez C. N. Meibers H. E. Pasare C. (2025). Bidirectional communication between the innate and adaptive immune systems. Annu. Rev. Immunol. 43 (1), 489–514. 10.1146/annurev-immunol-083122-040624 40279312 PMC12120936

[B89] Wrona M. V. Ghosh R. Coll K. Chun C. Yousefzadeh M. J. (2024). The 3 i's of immunity and aging: immunosenescence, inflammaging, and immune resilience. Front. Aging 5, 1490302. 10.3389/fragi.2024.1490302 39478807 PMC11521913

[B90] Yousefzadeh M. J. Flores R. R. Zhu Y. Schmiechen Z. C. Brooks R. W. Trussoni C. E. (2021). An aged immune system drives senescence and ageing of solid organs. Nature 594 (7861), 100–105. 10.1038/s41586-021-03547-7 33981041 PMC8684299

[B91] Zhang L. Pitcher L. E. Prahalad V. Niedernhofer L. J. Robbins P. D. (2022a). Targeting cellular senescence with senotherapeutics: senolytics and senomorphics. FEBS J. 290, 1362–1383. 10.1111/febs.16350 35015337

[B92] Zhang L. Pitcher L. E. Yousefzadeh M. J. Niedernhofer L. J. Robbins P. D. Zhu Y. (2022b). Cellular senescence: a key therapeutic target in aging and diseases. J. Clin. Invest 132 (15), e158450. 10.1172/JCI158450 35912854 PMC9337830

[B93] Zingoni A. Cecere F. Vulpis E. Fionda C. Molfetta R. Soriani A. (2015). Genotoxic stress induces senescence-associated ADAM10-Dependent release of NKG2D MIC ligands in multiple myeloma cells. J. Immunol. 195 (2), 736–748. 10.4049/jimmunol.1402643 26071561

